# Antimicrobial activity of a decapeptide against *Candida albicans*

**DOI:** 10.1128/spectrum.01197-25

**Published:** 2025-12-19

**Authors:** Zhongjie Li, Yabo Liu, Ye Yuan, Zhuoqian Sun, Jiao Zhang, Qi Dai, Shasha Li, Bo Deng, Wenlu Zhang, Yanfang Dong, Gaofeng Liang, Shegan Gao

**Affiliations:** 1 Henan Provincial Key Laboratory of Microbiota and Esophageal Cancer Prevention and Control, Henan University of Science and Technology 74623, Luoyang, China; 2 Microbial Pathogen and Anti-Infection Research Group, School of Basic Medicine and Forensic Medicine, Henan University of Science and Technology 74623, Luoyang, China; 3 Henan University of Science and Technology, The First Affiliated Hospital, Luoyang, China; Emory University School of Medicine, Atlanta, Georgia, USA

**Keywords:** *Candida albicans*, antimicrobial peptide, decapeptide, fungicidal

## Abstract

The increasing frequency of infections and drug resistance of *Candida albicans* has emerged as significant public health challenges, highlighting the urgent need for new antifungal agents. In this study, a decapeptide AntiCADP was designed, which could effectively inhibit the growth of *C. albicans*. AntiCADP killed *C. albicans* cells by disrupting the cell membrane, inducing ROS accumulation, damaging mitochondria, and ultimately leading to cellular necrosis. Additionally, AntiCADP inhibited the hyphal morphogenesis and biofilm formation of *C. albicans*. AntiCADP could also kill *C. albicans* cells in the mature biofilm. In a mouse subcutaneous infection model, AntiCADP significantly inhibited the development of abscesses, reduced *C. albicans* cell counts within abscesses, and suppressed inflammatory cell infiltration at the infected area. Taken together, AntiCADP has the potential to be an antifungal agent against skin infections caused by *C. albicans*.

**IMPORTANCE:**

To effectively cope with the increasing frequency of infections and drug resistance of *Candida albicans*, various types of new antimicrobial molecules have been studied. Among these molecules, antimicrobial peptides have attracted great attention. In the present study, we designed a decapeptide AntiCADP, which showed good anti-*C*. *albicans* activity *in vitro* and *in vivo*. AntiCADP killed *C. albicans* cells via multiple modes, including disrupting the cell membrane, inducing ROS accumulation, damaging mitochondria, and inducing cellular necrosis. AntiCADP could also inhibit hyphal morphogenesis and biofilm formation of *C. albicans* and kill *C. albicans* cells in the mature biofilm. Thus, AntiCADP had the potential against skin infections caused by *C. albicans*.

## INTRODUCTION

Candidiasis, a common fungal infection caused by *Candida* species, has become a global health problem, with *Candida albicans* being the predominant causative pathogen (1). As a commensal organism, *C. albicans* colonizes the surface of oral mucosae, gut mucosae, vaginal mucosae, and skin of most humans without causing disease (2). However, under specific conditions such as immunosuppression, diabetes, or antibiotic use, *C. albicans* can become pathogenic and cause a variety of diseases ranging from mucosal infections (e.g., cutaneous, oropharyngeal, and vulvovaginal candidiasis) to life-threatening systemic manifestations (e.g., candidemia and invasive candidiasis) (3, 4). Nowadays, due to the increasing antifungal resistance and immunocompromised populations, the frequency of *C. albicans* infections has increased significantly, leading to higher healthcare costs and mortality (5). Consequently, *C. albicans* is categorized within the critical priority group of fungal pathogens by the World Health Organization. Although traditional antifungal drugs remain the primary treatment for *C. albicans* infections, the development of new antifungal drugs is imperative.

During the exploration of new antimicrobial agents, antimicrobial peptides (AMPs) show great application potential. AMPs are short amino acid chains of peptides, which were initially discovered and isolated from various life forms (including animals, plants, and microorganisms) as defense molecules of the innate immune system (6). They show excellent activity against a broad spectrum of pathogens, including drug-resistant strains (7). Unlike traditional antibiotics, AMPs kill microbial cells via multifaceted mechanisms, including disruption of membrane and/or interaction with intracellular targets, making it difficult for pathogens to develop resistance to them (8, 9). AMPs usually share features, including positive charge, high hydrophobic residue content, and amphipathic conformation (10). These structural features enable the rational design of artificial AMPs and peptide derivatives (11). Currently, over 24,000 AMPs are recorded in the Collection of Anti-Microbial Peptides database (https://camp.bicnirrh.res.in/).

In this study, we designed a decapeptide (BTP10) derived from the first helical region (residues 1–10aa) of *Bos taurus* phospholipase A2 based on the characteristics of AMPs, which showed weak activity against *C. albicans*. Studies have shown that enhancing the net positive charge, hydrophobic moment, or hydrophobic residue proportion can improve the antimicrobial activity of AMPs (12). Due to the limited activity of BTP_10_ against *C. albicans*, we designed several peptide derivatives via amino acid substitution, among which the peptide derivative AntiCADP (anti*-C*. *albicans* decapeptide: KLWKFLKKIL) showed good anti-*C*. *albicans* activity *in vitro* and *in vivo*. Furthermore, the action mechanism of AntiCADP against *C. albicans* and the influence of AntiCADP on the virulence factors of *C. albicans* were investigated.

## RESULTS

### Peptides and *in vitro* anti-*C*. *albicans* activity

**Fig 1:**
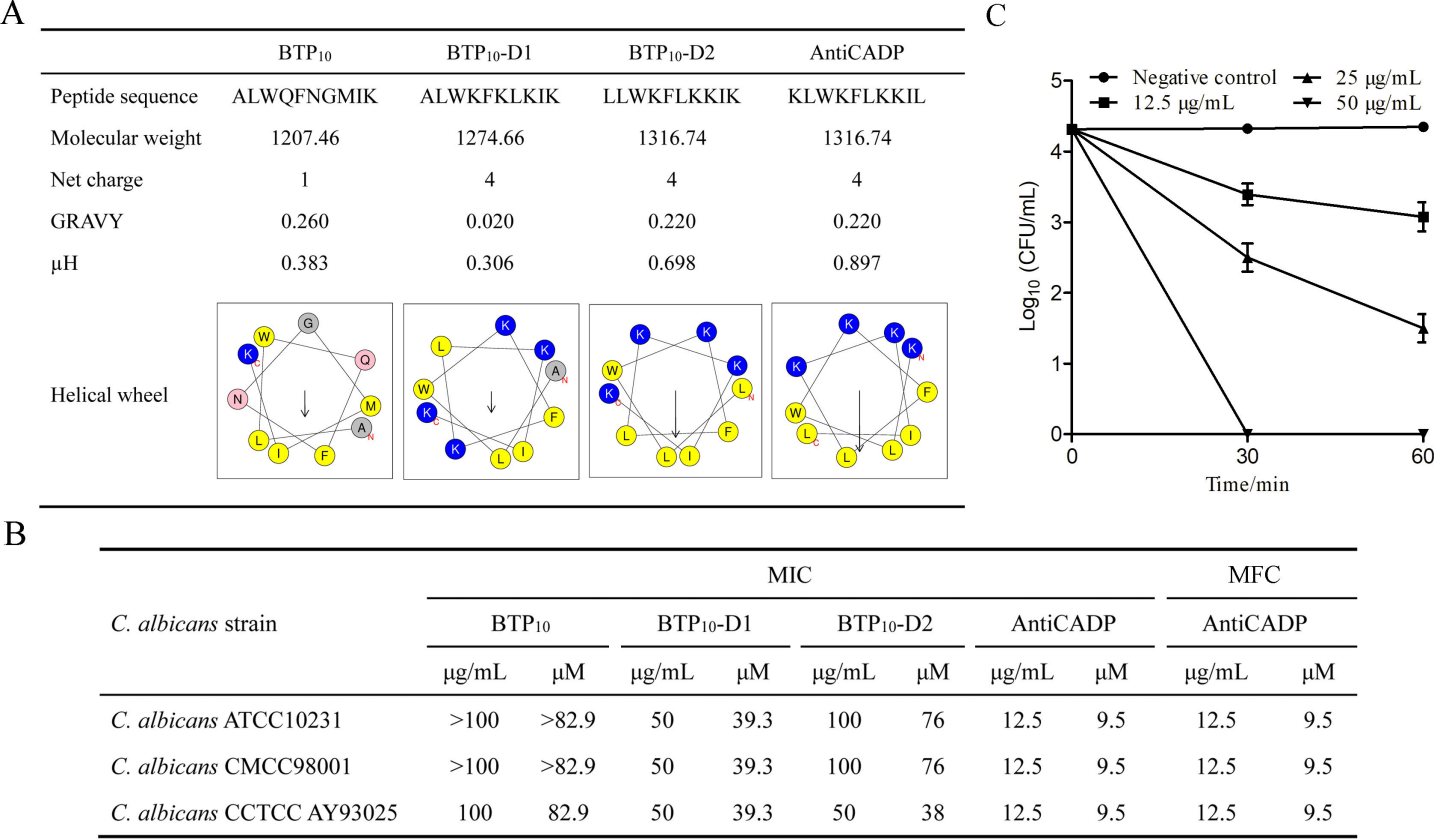
Peptides and activities. (**A**) Characteristics of the peptides. Molecular weight and GRAVY (grand average of hydropathicity) were determined by ProtParam (https://web.expasy.org/protparam/). µH (hydrophobic moment) and helical wheel diagram were determined by the Heliquest (https://heliquest.ipmc.cnrs.fr/cgi-bin/ComputParams.py). (**B**) *In vitro* anti-*C. albicans* activity of the peptides. (**C**) Time-killing kinetics assay. Negative control: 0.9% saline.

Based on the peptide BTP_10_, three peptide derivatives (BTP_10_-D1, BTP_10_-D2, and AntiCADP) were designed (Fig. 1A). BTP_10_-D1, BTP_10_-D2, and AntiCADP had an increased net positive charge compared to BTP_10_, and BTP_10_-D2 and AntiCADP had an increased hydrophobic moment (µH) compared to BTP_10_. Among these peptides, AntiCADP had the highest µH and could form a typical amphiphilic conformation (Fig. 1A). As shown in Fig. 1B, the MIC of BTP_10_ against *C. albicans* ATCC10231, *C. albicans* CMCC98001, and *C. albicans* CCTCC AY93025 was >100 µg/mL, >100 µg/mL, and 100 μg/mL, respectively. The activities of BTP_10_-D1 and BTP_10_-D2 against the tested *C. albicans* strains did not show any significant changes compared to that of BTP_10_, with the MICs of 50–100 μg/mL. The activity of AntiCADP against the tested *C. albicans* strains was significantly greater than that of BTP_10_, with the MICs of 12.5 μg/mL for all strains. Thus, AntiCADP was the most effective anti-*C*. *albicans* peptide among these peptides. Moreover, the MFC of AntiCADP against *C. albicans* ATCC10231, *C. albicans* CMCC98001, and *C. albicans* CCTCC AY93025 was consistent with the MIC (Fig. 1B), indicating AntiCADP is a fungicidal peptide.

### AntiCADP kills *C. albicans* cells in a dose- and time-dependent manner

To further evaluate the action mode of AntiCADP against *C. albicans*, the time-killing kinetics assay was performed. As shown in Fig. 1C, after treatment of *C. albicans* cells with AntiCADP at a concentration of 12.5 μg/mL for 30 and 60 min, the viable cells decreased by about 1 log. When *C. albicans* cells were treated with AntiCADP at a concentration of 25 μg/mL for 30 min and 60 min, the viable cells decreased by about 2-log and 3-log, respectively. No viable cells were observed after treatment of *C. albicans* cells with AntiCADP at a concentration of 50 μg/mL for 30 min. These results indicated that AntiCADP killed *C. albicans* cells in a dose- and time-dependent manner. These results further indicated that AntiCADP is a fungicidal peptide.

### AntiCADP damages the ultrastructure of *C. albicans*

**Fig 2:**
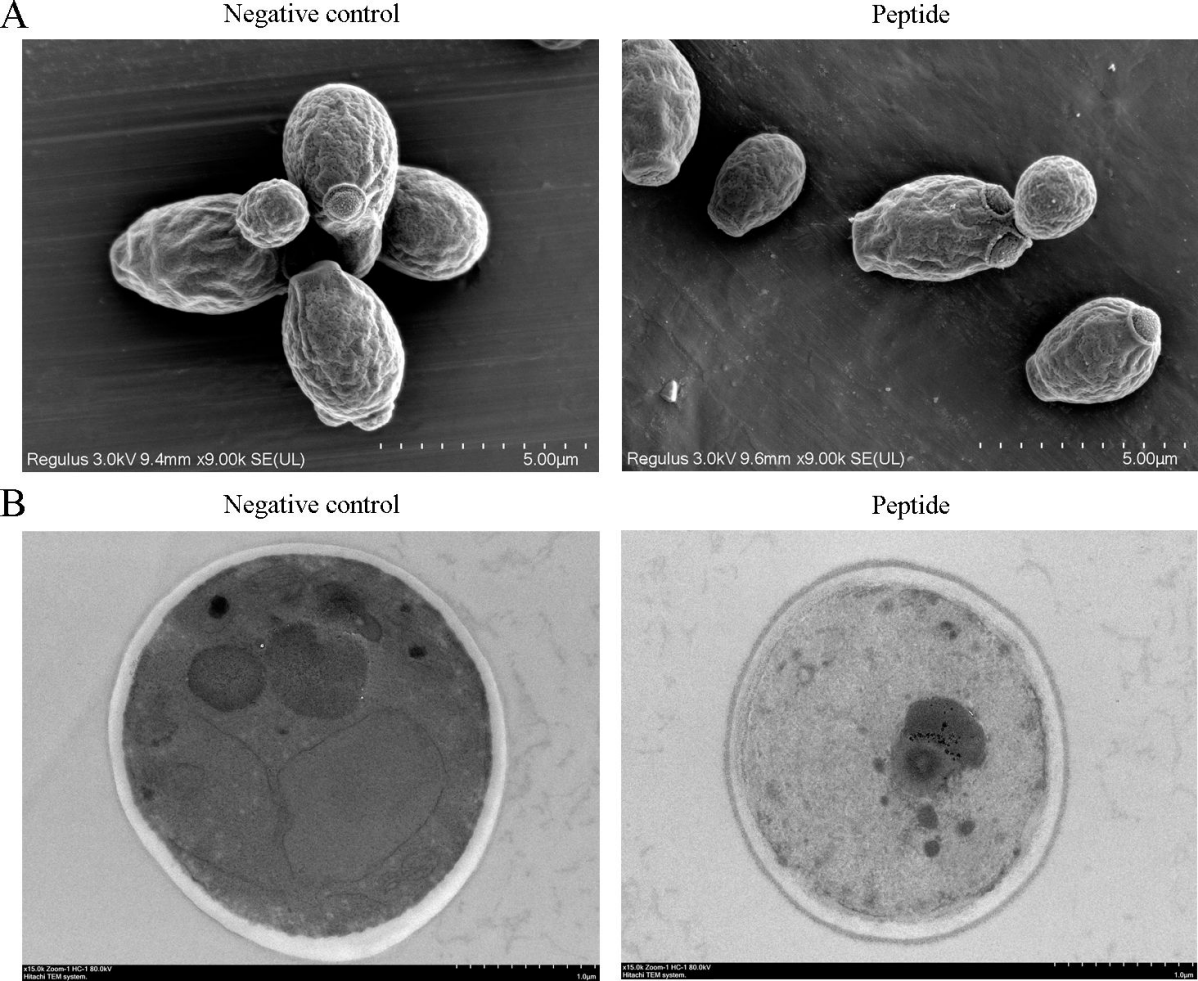
Electron microscopy detection. (**A**) Scanning electron microscope detection. (**B**) Transmission electron microscope detection. Negative control: 0.9% saline. The *C. albicans* cells were treated with AntiCADP at a final concentration of 50 μg/mL at 35°C for 30 min.

To evaluate the influence of AntiCADP on the morphology and ultrastructure of *C. albicans* cells, AntiCADP-treated *C. albicans* cells were detected using a scanning electron microscope and a transmission electron microscope. As shown in Fig. 2A, the morphology of *C. albicans* cells did not show any obvious changes after being treated with AntiCADP compared to the non-AntiCADP-treated cells. As shown in Fig. 2B, the boundary between the cell wall and the cell membrane, as well as the regions of the intracellular membrane structures, is clearly defined in the non-AntiCADP-treated cells, whereas the distinctness of the boundary between the cell wall and the cell membrane is reduced and the regions of the intracellular membrane structures are not visible in the AntiCADP-treated cells. It is most likely caused by the disruption of the cell membrane and intracellular membrane structures, leading to the leakage of cellular contents. These results indicated that AntiCADP did not disrupt the cell wall of *C. albicans* cells but might disrupt the membrane structures.

### AntiCADP damages the cell membrane of *C. albicans*

**Fig 3:**
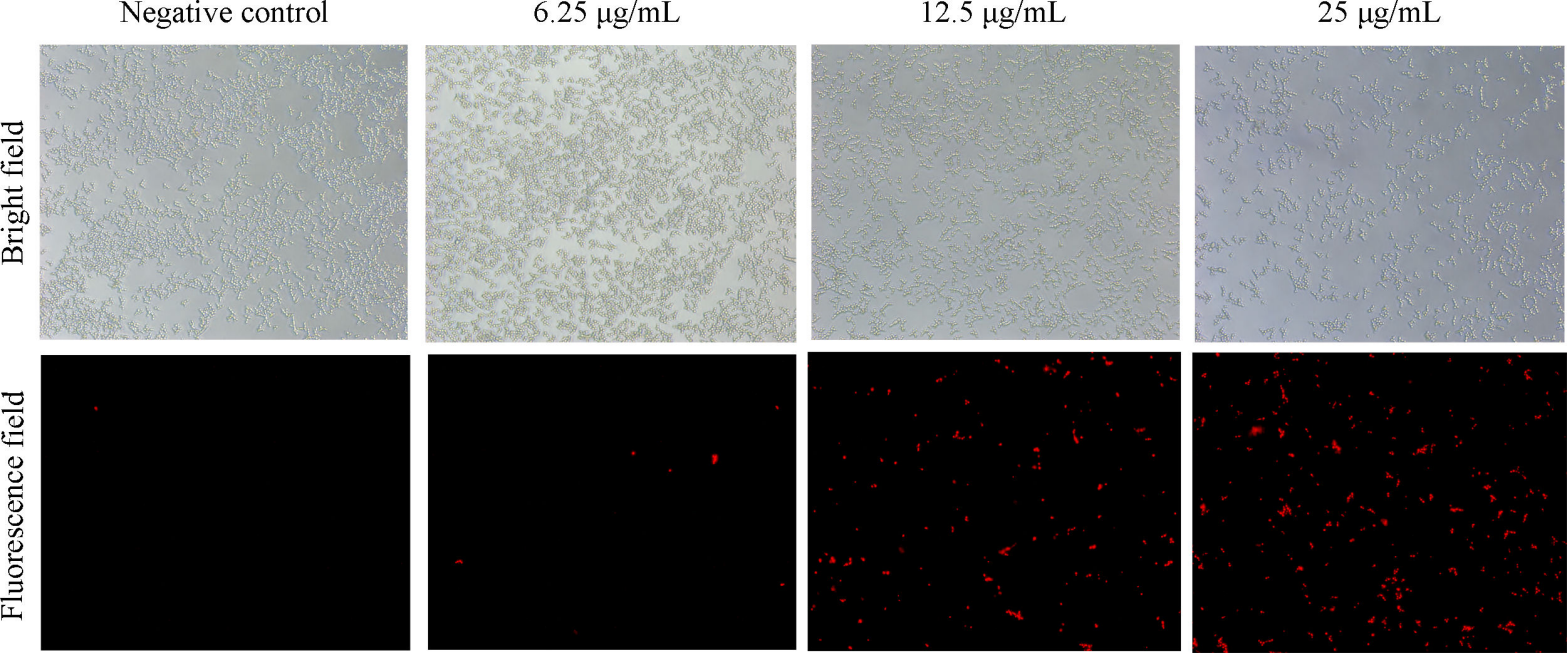
PI absorption assays. The *C. albicans* cells were treated with AntiCADP or a negative control at 35°C for 30 min and then incubated with PI at the final concentration of 10 μg/mL for 10 min. Negative control: 0.9% saline.

To evaluate the influence of AntiCADP on the membrane integrity of *C. albicans* cells, the PI absorption assay was performed. The fluorescent dye PI only penetrates cells with damaged membranes, binds to nucleic acids, and produces red fluorescence, which can be observed under a fluorescence microscope. As shown in Fig. 3, no fluorescence-positive cell was observed in the non-peptide treatment (negative control) group. After treating *C. albicans* with AntiCADP at the concentrations of 6.25 μg/mL, 12.5 μg/mL, and 25 μg/mL, respectively, the number of fluorescence-positive cells increased along with the peptide concentration. These results demonstrated that AntiCADP damaged the cell membrane of *C. albicans* cells.

### AntiCADP increases ROS production in *C. albicans*

**Fig 4:**
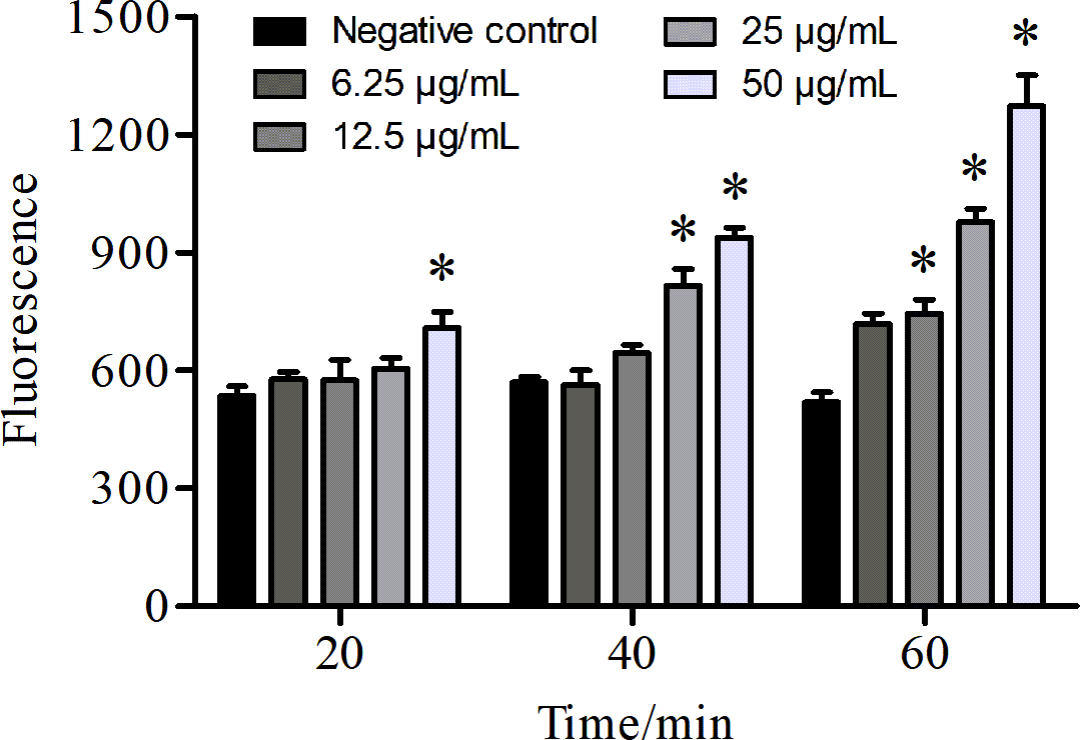
ROS measurement. The *C. albicans* cells were incubated with DCFH-DA at 35°C at the final concentration of 10 μg/mL for 10 min and treated with AntiCADP or a negative control for 20 min, 40 min, or 60 min, respectively. The fluorescence was measured at the excitation and emission wavelengths of 488 and 525 nm, respectively. Negative control: 0.9% saline. *P < 0.05.

To evaluate the influence of AntiCADP on the production of ROS in *C. albicans* cells, the fluorescence dye DCFH-DA was used. As shown in Fig. 4, there was an increase in the fluorescence intensity after treating *C. albicans* cells with AntiCADP. Moreover, the fluorescence intensity increased with the increase of the peptide concentration and action time. These results indicated that AntiCADP could lead to ROS generation and accumulation in *C. albicans* cells.

### AntiCADP decreases the mitochondrial membrane potential of *C. albicans*

**Fig 5:**
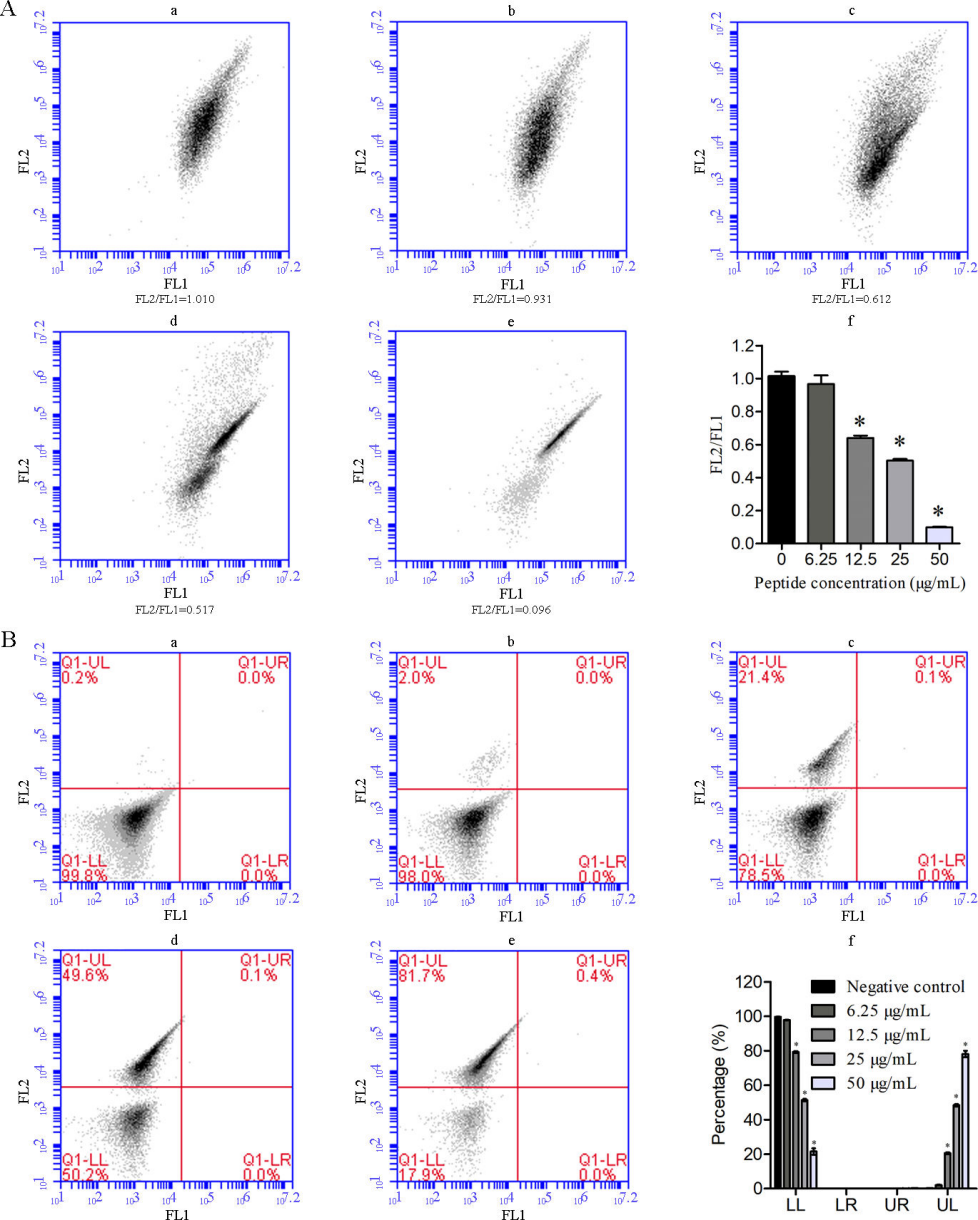
Flow cytometry assays. (**A**) Mitochondrial membrane potential assays. (a–e) Results of flow cytometry analysis. (f) Percentage of DCFH-DA stained cells. FL1: green fluorescence intensity of JC-1 monomer; FL2: red fluorescence intensity of JC-1 aggregates. (**B**) Apoptosis/necrosis detection assays. (a–e) Results of flow cytometry analysis. (f) Percentage of normal, early apoptosis, late apoptosis, and necrosis cells. FL1: annexin V-FITC; FL2: PI. Negative control: 0.9% saline. *P < 0.05.

To evaluate the influence of AntiCADP on the mitochondria of *C. albicans* cells, the mitochondrial membrane potential was investigated using flow cytometry. As a mitochondrial membrane potential-dependent fluorescent dye, JC-1 forms aggregates at high mitochondrial membrane potential state, producing red fluorescence and exists in monomeric form at low mitochondrial membrane potential, producing green fluorescence. Therefore, a decrease in the ratio of red/green fluorescence intensity represents mitochondrial depolarization. As shown in Fig. 5A, FL2 represents fluorescence intensities of aggregates JC-1, and FL1 represents fluorescence intensities of monomer JC-1. The FL2/FL1 ratio decreased in a dose-dependent manner after treating *C. albicans* cells with AntiCADP (Fig. 5Af), indicating a decrease in the mitochondrial membrane potential. These results indicated that AntiCADP could lead to depolarization of the mitochondrial membrane in *C. albicans* cells.

### AntiCADP induces necrosis of *C. albicans* cells

To evaluate whether AntiCADP induces cell apoptosis and/or necrosis in *C. albicans*, *C. albicans* ATCC10231 cells were analyzed using flow cytometry after treatment with AntiCADP or not. As shown in Fig. 5B, the percentage of early apoptosis cells (quadrant Q1-LR) and late apoptosis cells (quadrant Q1-UR) did not show any obvious changes after treatment with AntiCADP compared to those of the negative control group. However, the percentage of necrosis cells (quadrant Q1-UL) increased with the increase of AntiCADP concentration. These results indicated that AntiCADP did not induce apoptosis in *C. albicans* cells but necrosis.

### AntiCADP inhibits hyphal formation of *C. albicans*

**Fig 6:**
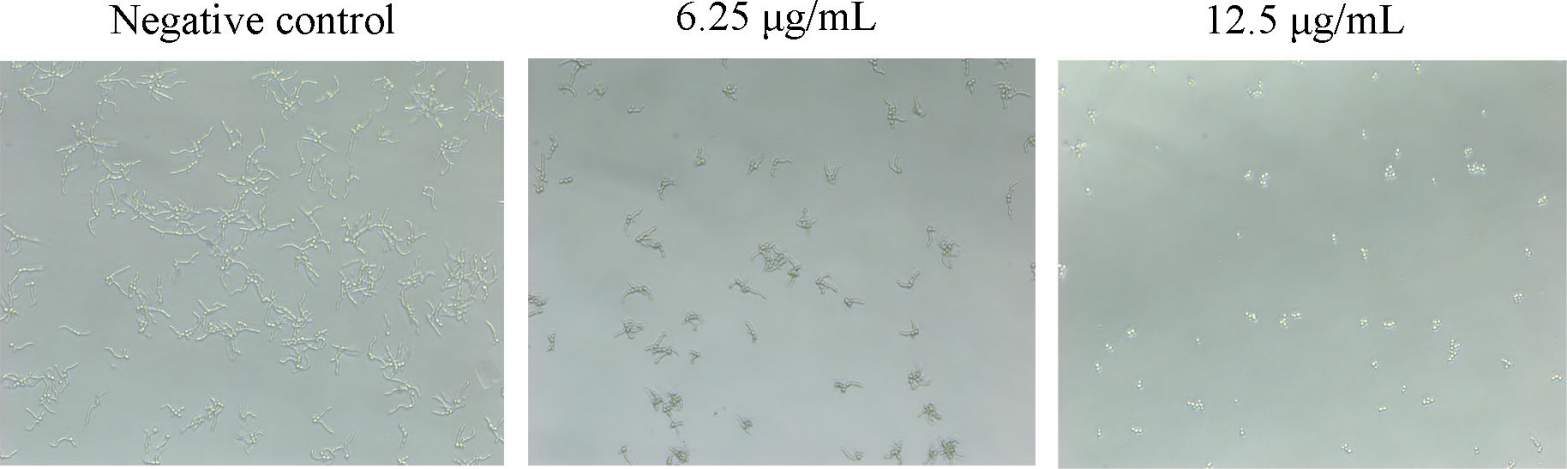
Hyphal formation. The *C. albicans* cells were treated with AntiCADP or a negative control at 35°C for 4 h and then observed using a microscope. Negative control: 0.9% saline.

To evaluate the influence of AntiCADP on the virulence factors of *C. albicans*, hyphal formation *in C. albicans* ATCC10231 cells was investigated after treatment with AntiCADP or without it. As shown in Fig. 6, *C. albicans* cells formed long hyphae in the non-peptide treatment group (negative control). When *C. albicans* cells were treated with AntiCADP at a concentration of 6.25 μg/mL, the number of cells forming hyphae and the length of the hyphae were significantly decreased. When *C. albicans* cells were treated with AntiCADP at the concentration of 12.5 μg/mL, no cells could form hyphae. These results indicated that AntiCADP inhibited the yeast-to-hypha transition in *C. albicans* cells.

### AntiCADP affects the biofilm of *C. albicans*

**Fig 7:**
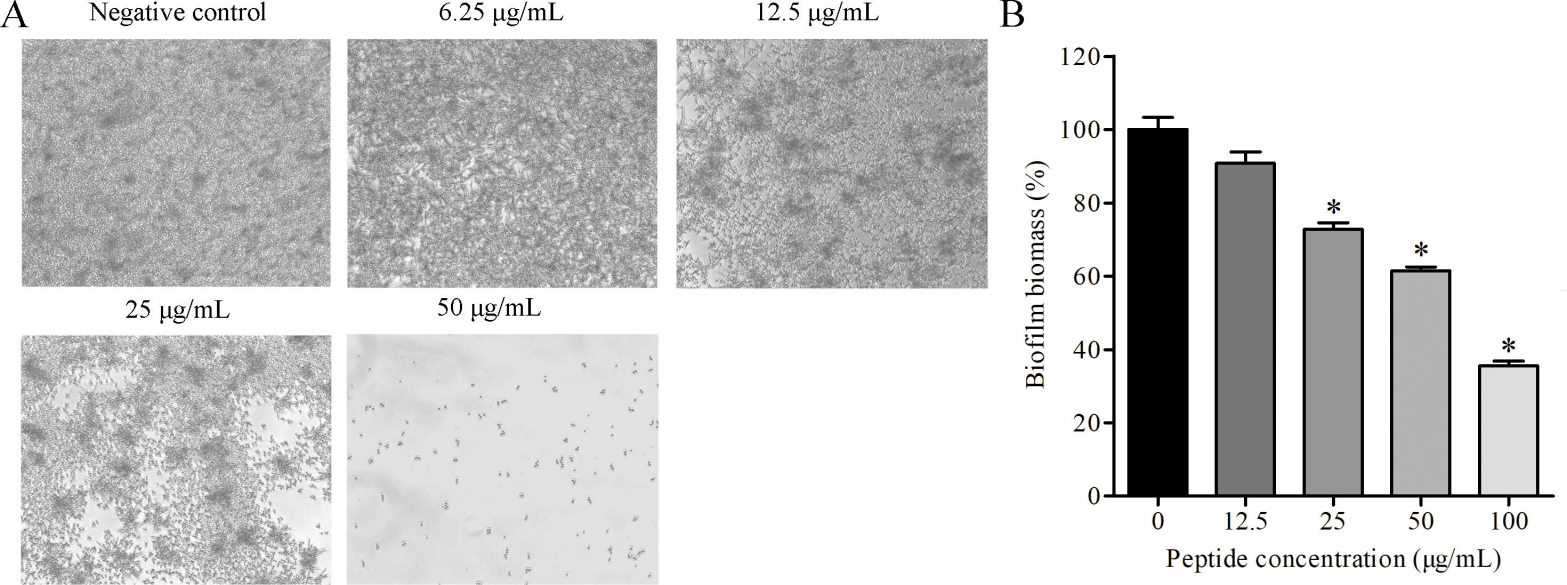
Influence on biofilm. (**A**) Inhibitory effects of AntiCADP on biofilm formation were investigated using a microscope. (**B**) Inhibitory effects of AntiCADP on mature biofilms were investigated by XTT assay. Negative control: 0.9% saline. *P < 0.05.

To further evaluate the influence of AntiCADP on the virulence factors of *C. albicans*, biofilm formation of *C. albicans* ATCC10231 cells was investigated after treatment with AntiCADP or not. As shown in Fig. 7A and C. *albicans* cells formed dense biofilm in the non-peptide treatment group (negative control). When *C. albicans* cells were treated with AntiCADP, both the biofilm density and the hyphal formation were decreased, and no biofilm formed after treatment of *C. albicans* cells with AntiCADP at a concentration of 50 μg/mL. Moreover, AntiCADP significantly reduced the biomass of the mature biofilms of *C. albicans* (Fig. 7B). These findings indicated that AntiCADP not only inhibited the biofilm formation but also reduced the mature biofilms of *C. albicans*.

### AntiCADP has low toxicity

To evaluate the toxicity of AntiCADP, hemolysis assay and acute toxicity assay were performed. The hemolysis assay showed that only 1.39%, 5.21%, and 16.67% RBCs were hemolyzed by AntiCADP at the concentrations of 25 μg/mL, 50 μg/mL, and 100 μg/mL, respectively. In the acute toxicity assay, all the mice treated with AntiCADP by intraperitoneal injection did not show any adverse events and survived in a seven-day study period with the dose up to 40 mg/kg. These results indicated that AntiCADP had low toxicity.

### AntiCADP inhibits *C. albicans in vivo*

**Fig 8:**
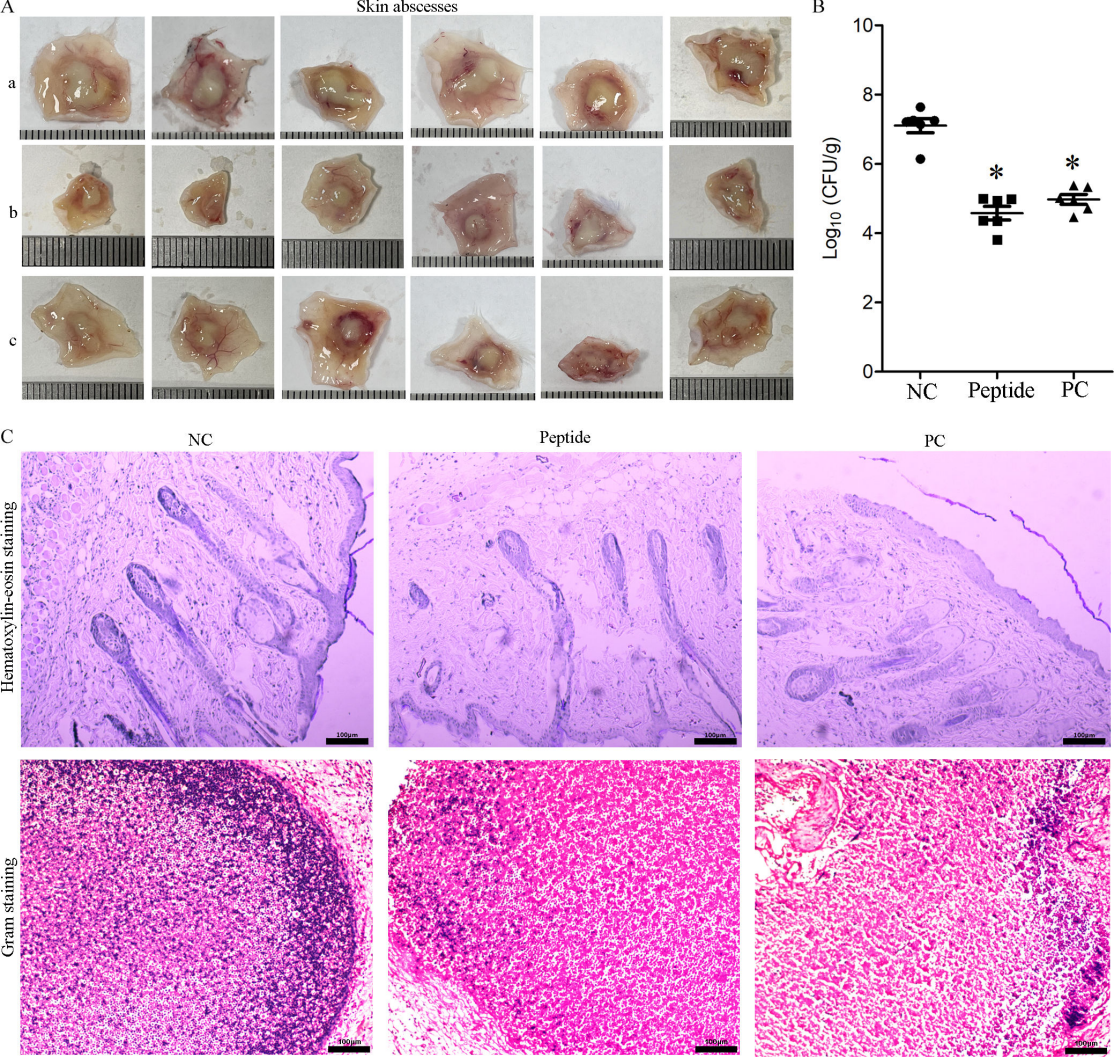
*In vivo* anti-*C. albicans* activity of AntiCADP. (**A**) Abscesses from each group. (a) Abscesses from the negative control group. (b) Abscesses from the peptide treatment group. (c) Abscesses from the positive control group. Each small division on the scale is 1 mm. (**B**) CFU per gram of the tissue. NC: negative control, 0.9% saline. PC: positive control, clotrimazole. *P < 0.05. (**C**) Hematoxylin-eosin and Gram staining. Bar: 100 μm. NC: negative control, 0.9% saline. PC: positive control, clotrimazole.

To evaluate the *in vivo* anti-*C*. *albicans* activity of AntiCADP, a mouse subcutaneous infection model was established. As shown in Fig. 8, the mice infected with *C. albicans* in the non-peptide treatment group (negative control) formed large skin abscesses (Fig. 8Aa). However, skin abscesses in the peptide treatment group (Fig. 8Ab) and the Clotrimazole treatment group (positive control group, Fig. 8Ac) were significantly smaller compared to those of the non-peptide treatment group (Fig. 8Aa). Furthermore, treatment with AntiCADP and clotrimazole significantly decreased both *C. albicans* cell counts in skin abscesses (Fig. 8B and C) and inflammatory infiltration in the infection area (Fig. 8C). These results indicated that AntiCADP had good anti-*C*. *albicans* activity *in vivo*.

## DISCUSSION

As the most predominant human opportunistic pathogen, *C. albicans* has gained increasing attention due to the increase in frequency, healthcare costs, and mortality of infections (5). Moreover, drug resistance in *C. albicans* has continuously increased in recent years (13). Thus, the need for novel and more effective anti-*C*. *albicans* agents is urgent, and AMPs have been a good choice (14). In this study, we designed a decapeptide AntiCADP (KLWKFLKKIL), which was a fungicidal peptide that could effectively inhibit the growth of *C. albicans in vitro* (Fig. 1).

AMPs kill or block the growth of microbial cells via multiple mechanisms, including disruption of membrane and/or interaction with intracellular targets (8, 9). Results from scanning electron microscope and transmission electron microscope detection showed that AntiCADP did not disrupt the cell wall of *C. albicans* cells but might disrupt the membrane structures (Fig. 2). Since AntiCADP is a fungicidal peptide, it kills microbial cells primarily by forming pores in the cytoplasmic membrane, leading to the dysfunction and depolarization of the cytoplasmic membrane, and ultimately inducing cell death (15). To verify whether AntiCADP could form pores in the cytoplasmic membrane of *C. albicans* cells, the PI absorption assay was performed. The results showed that AntiCADP enhanced the absorption of PI in *C. albicans* cells (Fig. 3), indicating the pore-forming action mode of AntiCADP. Thus, AntiCADP was also a membrane-active peptide. Besides, AMPs can induce the generation and accumulation of ROS, a key factor responsible for fungicidal effects, such as mellitin (16), Phibilin (17), and Histatin 5 (18). Therefore, it was investigated whether AntiCADP could induce the generation and accumulation of ROS in *C. albicans* cells. The results showed that AntiCADP could lead to ROS generation and accumulation in *C. albicans* cells in a dose- and time-dependent way (Fig. 4). High levels of ROS can lead to oxidative stress and cause mitochondrial injury (19). The stability of mitochondrial membrane potential is beneficial for maintaining normal physiological functions of cells, while a decrease in mitochondrial membrane potential leads to cellular physiological dysfunction. Our results showed that AntiCADP could lead to depolarization of the mitochondrial membrane of *C. albicans* cells (Fig. 5A). Besides mitochondrial injury, high levels of ROS can also cause impairment of normal metabolism, leading to cell apoptosis, necroptosis, and ferroptosis (20). Fungicidal drugs and fungicidal AMPs can kill fungal cells via ROS-related apoptosis and/or necrosis (21–27). Our results showed that AntiCADP did not induce apoptosis in *C. albicans* cells but rather necrosis (Fig. 5B). Thus, as a fungicidal peptide, AntiCADP killed *C. albicans* cells by disrupting the cell membrane, damaging the mitochondria, and inducing ROS-related cell necrosis.

*C. albicans* is a dimorphic fungus, switching morphology between yeast form and hyphal form (28). The yeast form is important for the dissemination of *C. albicans* via the bloodstream, while the hyphal form is beneficial for the adhesion and invasion of *C. albicans* to the host (29, 30). Moreover, the hyphal form enables *C. albicans* to escape from and destroy macrophages (31). Our results showed that AntiCADP could inhibit hyphal formation of *C. albicans* cells (Fig. 6). Thus, besides the direct fungicidal effects, AntiCADP had the potential to interrupt *C. albicans* infection by inhibiting the yeast-to-hyphal transition. The hyphal form is also required for *C. albicans* to establish biofilm, which is another critical pathogenic factor of *C. albicans*, hard to eradicate, resistant to host defenses, and conventional antifungal agents, leading to high morbidity and mortality rates (32, 33). Our results showed that AntiCADP could inhibit the biofilm formation of *C. albicans* and hyphal formation in the biofilm (Fig. 7A). Moreover, AntiCADP could kill C. albicans cells in the mature biofilm (Fig. 7B). Thus, AntiCADP could affect the formation of biofilms by interrupting the yeast-to-hyphal transition and has the potential to prevent *C. albicans* infection by inhibiting biofilm formation.

To further evaluate the potential application of AntiCADP, the *in vivo* activity of AntiCADP against *C. albicans* was evaluated by a mouse subcutaneous infection model. The results showed that AntiCADP not only significantly prevented the formation of abscesses but also significantly decreased the cell counts of *C. albicans* in abscesses and inhibited inflammatory infiltration (Fig. 8). Thus, AntiCADP showed good anti-*C*. *albicans* under physiological conditions, indicating the potential as an antifungal agent against skin infections caused by *C. albicans*.

Taken together, the peptide AntiCADP could effectively inhibit the growth of *C. albicans*. AntiCADP killed *C. albicans* cells by disrupting the cell membrane, inducing ROS accumulation, damaging mitochondria, and leading to cell necrosis. Moreover, AntiCADP inhibited the hyphal formation and biofilm formation of *C. albicans*. Furthermore, AntiCADP had good anti-*C*. *albicans* activity in the mouse subcutaneous infection model. Thus, AntiCADP had the potential as an antifungal agent against skin infections caused by *C. albicans*.

## MATERIALS AND METHODS

### Peptide, fungal strains, and animals

The C-terminally amidated peptides used in this study were synthesized by GL Biochem (Shanghai, China) and had a purity of over 95%. *C. albicans* ATCC10231, *C. albicans* CMCC 98001, and *C. albicans* CCTCC AY93025 used in this study were cultured using Yeast Extract Peptone Dextrose (YPD) medium. Female BALB/c mice (18–21 g) were obtained from Henan SKBEX Biology Co., Ltd., and the China Science and Technology Resource identification number for the mice was CSTR:15497.09.N000009 (https://nrla.nifdc.org.cn/nrla/index.html). The mice were maintained under standard conditions of humidity (50% ± 5%), temperature (25 ± 2°C), and dark-light cycles (12 h each) with free access to food and water.

### Antimicrobial activity

The antimicrobial activity of the peptides against *C. albicans* was performed using the broth microdilution assay according to the Clinical and Laboratory Standards Institute guidelines (34). Briefly, the peptides were serially diluted in 0.9% saline, and the *C. albicans* cells were diluted in YPD medium to 10^3^–10^4^ cells/mL. Then, 40 μL of the peptide dilution and 160 μL of the *C. albicans* cell suspension were added into sterile 96-well cell culture plates. The plates were incubated with continuous shaking at 200 rpm at 35°C for 18–24 h. The lowest concentration at which no *C. albicans* cell growth was detected was determined as the minimum inhibitory concentration (MIC). Furthermore, broth from the wells with no visible growth of *C. albicans* was plated onto YPD agar plates, and the lowest peptide concentration that caused a ≥99.9% reduction in the initial cell count was determined as the minimum fungicidal concentration (MFC). The experiments were repeated at least three times.

### Time-killing kinetics

The *in vitro* killing kinetics of AntiCADP against *C. albicans* were determined using the method described previously (17). Briefly, *C. albicans* ATCC10231 cells (10^4^–10^5^ cells/mL) were treated with AntiCADP at the final concentrations of 12.5 μg/mL, 25 μg/mL, or 50 μg/mL, respectively, and no peptide treatment was served as a negative control. Then, aliquots were collected at 30 min and 60 min and then plated on the YPD agar plates. After these plates were incubated at 35°C for 18–24 h, CFU was counted, and time-killing kinetics were calculated.

### Electron microscopy

A scanning electron microscope and a transmission electron microscope were used to observe the morphological and ultrastructural changes in AntiCADP-treated *C. albicans* cells, respectively. Briefly, *C. albicans* ATCC10231 cells were diluted in YPD medium to approximately 10^6^ cells/mL. Then, the *C. albicans* cell suspension was treated with AntiCADP at a final concentration of 50 μg/mL at 35°C for 30 min. After incubation, the cells were washed with PBS and fixed with 2.5% glutaraldehyde in PBS. For scanning electron microscope detection, the fixed cells were dehydrated with increasing concentrations of ethanol (30, 50, 70, 90, and 100%) and air-dried at room temperature. Then, the dry samples were gold-coated using a Hitachi MC1000 sputter coater and then observed with a Hitachi Regulus 8100 scanning electron microscope. For transmission electron microscope detection, the fixed cells were stained with 1% OsO4 solution, dehydrated with increasing concentrations of ethanol (30, 50, 70, 90, and 100%), embedded in epoxy resins, and then semi-thin sectioned and examined using a Hitachi HT7800 transmission electron microscope.

### Membrane integrity assay

The influence of AntiCADP on the cell membrane integrity of *C. albicans* cells was determined using the PI absorption assay (35). Briefly, *C. albicans* ATCC10231 cells were diluted in YPD medium to approximately 10^6^ cells/mL. Then, the *C. albicans* cell suspension was treated with AntiCADP at the final concentrations of 6.25 μg/mL, 12.5 μg/mL, and 25 μg/mL, respectively, and no peptide treatment was served as a negative control. After incubation at 35°C for 30 min, the *C. albicans* cells were harvested by centrifugation, washed, and resuspended in 0.9% saline. Thereafter, the *C. albicans* cells were incubated with PI at the final concentration of 10 μg/mL for 10 min. Then, the *C. albicans* cells were harvested by centrifugation, washed, resuspended in 0.9% saline, and observed using a fluorescence microscope. The experiment was repeated at least three times.

### ROS measurement

The influence of AntiCADP on ROS generation and accumulation in *C. albicans* cells was determined using a fluorescence detection assay (36). Briefly, *C. albicans* ATCC10231 cells were harvested by centrifugation, washed with, and resuspended in 0.9% saline to approximately 10^6^ cells/mL, and then incubated with DCFH-DA at 35°C at the final concentration of 10 μg/mL for 10 min. After incubation, 50 μL of the *C. albicans* cell resuspension and 50 μL of AntiCADP dilution of different concentrations were added into a Costar 96-well flat-bottom black plate. The final concentration of AntiCADP was 6.25 μg/mL, 12.5 μg/mL, 25 μg/mL, and 50 μg/mL, respectively. Each concentration was conducted in triplicate, and no peptide treatment served as a negative control. The fluorescence was measured at the excitation and emission wavelengths of 488 and 525 nm, respectively. The experiment was repeated at least three times.

### Mitochondrial membrane potential assays

The influence of AntiCADP on mitochondrial membrane potential of *C. albicans* cells was determined using flow cytometry (37). Briefly, *C. albicans* ATCC10231 cells were diluted in YPD medium to approximately 10^6^ cells/mL. Then, the *C. albicans* cell suspension was treated with AntiCADP at the final concentrations of 6.25 μg/mL, 12.5 μg/mL, 25 μg/mL, and 50 μg/mL, respectively, and no peptide treatment was served as a negative control. After incubation at 35°C for 2 h, the *C. albicans* cells were harvested by centrifugation, washed with PBS, and resuspended in PBS. Then, the *C. albicans* cells were treated with the Mitochondrial Membrane Potential Assay Kit (Solarbio Life Science, M8650). Thereafter, the cells were analyzed by flow cytometry, and the ratio of the fluorescence intensities of aggregates JC-1 to monomer was calculated. The experiment was repeated at least three times.

### Apoptosis/necrosis detection assays

The influence of AntiCADP on apoptosis/necrosis of *C. albicans* cells was determined using flow cytometry (17). Briefly, *C. albicans* ATCC10231 cells were diluted in YPD medium to approximately 10^6^ cells/mL. Then, the *C. albicans* cell suspension was treated with AntiCADP at the final concentrations of 6.25 μg/mL, 12.5 μg/mL, 25 μg/mL, and 50 μg/mL, respectively, and no peptide treatment was served as a negative control. After incubation at 35°C for 2 h, the *C. albicans* cells were harvested by centrifugation, washed with, and resuspended in PBS. Then, the *C. albicans* cells were treated with Annexin V-FITC Apoptosis Detection Kit (Solarbio Life Science, CA1020). Thereafter, the cells were analyzed by flow cytometry. The experiment was repeated at least three times.

### Hypha formation assays

The influence of AntiCADP on hyphae formation of *C. albicans* cells was determined using a microscope (38). Briefly, *C. albicans* ATCC10231 cells were harvested by centrifugation, washed with 0.9% saline, and resuspended in RPMI 1640 medium (supplemented with 10% fetal bovine serum) to approximately 10^6^ cells/mL. Then, 100 μL of the *C. albicans* cell suspension and 100 μL of the peptide dilution of different concentrations were added into sterile 96-well cell culture plates. The final concentration of the peptide was 6.25 μg/mL and 12.5 μg/mL, respectively, and no peptide treatment served as a negative control. After incubation at 35°C for 4 h, the *C. albicans* cells were observed using a microscope. The experiment was repeated at least three times.

### Influence on biofilm

The influence of AntiCADP on biofilm formation of *C. albicans* cells was determined using a microscope (39). Briefly, *C. albicans* ATCC10231 cells were harvested by centrifugation, washed with 0.9% saline, and resuspended in RPMI 1640 medium (supplemented with 10% fetal bovine serum) to approximately 10^6^ cells/mL. Then, 100 μL of the *C. albicans* cell suspension and 100 μL of the peptide dilution of different concentrations were added into sterile 96-well cell culture plates. The final concentration of the peptide was 6.25 μg/mL, 12.5 μg/mL, 25 μg/mL, and 50 μg/mL, respectively, and no peptide treatment was served as a negative control. After incubation at 35°C for 18–24 h, the formation of biofilm was observed using a microscope. The experiment was repeated at least three times.

To evaluate the influence of AntiCADP on mature biofilm of *C. albicans* cells, the cell viability of the biofilms was measured using an XTT assay (40). Briefly, *C. albicans* ATCC10231 cells were harvested by centrifugation, washed with 0.9% saline, and resuspended in RPMI 1640 medium (supplemented with 10% fetal bovine serum) to approximately 10^6^ cells/mL. Then, 200 μL of the *C. albicans* cell suspension was added into sterile 96-well cell culture plates. After incubation at 35°C for 24 h, different concentrations of the peptide were added. The final concentration of the peptide was 12.5 μg/mL, 25 μg/mL, 50 μg/mL, and 100 μg/mL, respectively, and no peptide treatment was served as a negative control. After incubation at 35°C for 0.5 h, the cell viability of the biofilms was measured using the XTT assay.

### Toxicity assays

The *in vitro* toxicity of AntiCADP was evaluated using a hemolysis test. Briefly, freshly isolated red blood cells (RBCs) from normal mice blood samples were washed and resuspended in 0.9% saline to a concentration of 2% (vol/vol). Then, 100 μL of the RBCs suspension and 100 μL of the peptide dilution of different concentrations were added into sterile 96-well cell culture plates. A 1% Triton X-100 treatment served as the positive control, and no peptide treatment served as the negative control. After incubation with continuous shaking at 100 rpm at 37°C for 1 h, the plate was centrifuged at 1,000 × *g* for 10 min. Thereafter, the supernatant from each well (100 μL) was transferred to a new 96-well plate, and the absorbance of the supernatant was measured at 490 nm. The hemolysis percentage was calculated using the following equation: Hemolysis % = (*H*_sample_ − *H*_negative_)/(*H*_positive_ − *H*_negative_) × 100%, where *H* represents absorbance at 490 nm. The experiment was repeated at least three times.

The *in vivo* toxicity of AntiCADP was evaluated using an acute toxicity test in mice. Briefly, AntiCADP was dissolved and serially diluted in 0.9% saline. Then, the peptide dilution was given to each mouse by intraperitoneal injection at the designated doses (5 mg/kg, 10 mg/kg, 20 mg/kg, or 40 mg/kg) in 0.5 mL, with six mice per group. After treatment, the physiological status, weight, and mortality rate of the mice in each group were monitored once daily for 7 days.

### Mouse cutaneous infection model

The *in vivo* activity of AntiCADP against *C. albicans* was evaluated using a subcutaneous infection model in mice (41). Briefly, *C. albicans* ATCC10231 cells were harvested by centrifugation, washed with 0.9% saline, and resuspended in 0.9% saline to approximately 10^8^ cells/mL. Then, 50 μL of the *C. albicans* cell suspension was subcutaneously injected into the back of each mouse. These infected mice were randomly divided into three groups: peptide treatment group (AntiCADP, 500 μg/mL in 0.9% saline), non-peptide treatment group (negative control, 0.9% saline), and anti-fungal agent treatment group (positive control, clotrimazole, 500 μg/mL in 0.9% saline), six mice per group. One hour after infection, each mouse was subcutaneously injected with 50 μL of the corresponding agent solution into the infection area. The treatment was done once daily for 3 days, and all the mice were humanely euthanized on day 4. The whole skin abscess from each mouse was collected, one-half of the tissue was homogenized and serially diluted in 0.9% saline, and another half of the tissue was fixed with 4% paraformaldehyde. Thereafter, the dilutions were cultured on YPD agar plates at 35°C for 18–24 h, and the CFU per gram of the tissue (CFU/g) was calculated. The fixed tissues were used for hematoxylin-eosin staining and Gram staining. Hematoxylin-eosin staining was used to investigate the infiltration of inflammatory cells in the skin around the abscess, while Gram staining was used to investigate the yeast colonization in the abscess.

### Statistical analysis

The data were analyzed using the GraphPad Prism 6 software, and the differences between groups were analyzed using one-way ANOVA.

## Data Availability

Data will be made available on request.
